# The modified Glasgow prognostic score in prostate cancer: results from a retrospective clinical series of 744 patients

**DOI:** 10.1186/1471-2407-13-292

**Published:** 2013-06-17

**Authors:** Kashif Shafique, Michael J Proctor, Donald C McMillan, Hing Leung, Karen Smith, Billy Sloan, David S Morrison

**Affiliations:** 1 Institute of Health & Wellbeing, Public Health, University of Glasgow, 1 Lilybank Gardens, Glasgow G12 8RZ, UK; 2 Department of Community Medicine, Dow Medical College, Dow University of Health Sciences, Karachi, Pakistan; 3 University Department of Surgery, Faculty of Medicine, University of Glasgow, Royal Infirmary, Glasgow G31 2ER, UK; 4 Urology Department, Gartnavel General Hospital, 1053 Great Western Road, Glasgow G12 0YN, UK; 5 Beatson Institute for Cancer Research, Garscube Estate Switchback Road Bearsden, Glasgow G61 1BD, UK; 6 Department of Clinical Biochemistry, Royal Infirmary, Glasgow G4 0SF, UK; 7 West of Scotland Cancer Surveillance Unit, University of Glasgow, 1 Lilybank Gardens, Glasgow G12 8RZ, UK

**Keywords:** mGPS, Prostate cancer, Prognosis, PSA

## Abstract

**Background:**

As the incidence of prostate cancer continues to rise steeply, there is an increasing need to identify more accurate prognostic markers for the disease. There is some evidence that a higher modified Glasgow Prognostic Score (mGPS) may be associated with poorer survival in patients with prostate cancer but it is not known whether this is independent of other established prognostic factors. Therefore the aim of this study was to describe the relationship between mGPS and survival in patients with prostate cancer after adjustment for other prognostic factors.

**Methods:**

Retrospective clinical series on patients in Glasgow, Scotland, for whom data from the Scottish Cancer Registry, including Gleason score, Prostate Specific Antigen (PSA), C-reactive protein (CRP) and albumin, six months prior to or following the diagnosis, were included in this study.

The mGPS was constructed by combining CRP and albumin. Five-year and ten-year relative survival and relative excess risk of death were estimated by mGPS categories after adjusting for age, socioeconomic circumstances, Gleason score, PSA and previous in-patient bed days.

**Results:**

Seven hundred and forty four prostate cancer patients were identified; of these, 497 (66.8%) died during a maximum follow up of 11.9 years. Patients with mGPS of 2 had poorest 5-year and 10-year relative survival, of 32.6% and 18.8%, respectively. Raised mGPS also had a significant association with excess risk of death at five years (mGPS 2: Relative Excess Risk = 3.57, 95% CI 2.31-5.52) and ten years (mGPS 2: Relative Excess Risk = 3.42, 95% CI 2.25-5.21) after adjusting for age, socioeconomic circumstances, Gleason score, PSA and previous in-patient bed days.

**Conclusions:**

The mGPS is an independent and objective prognostic indicator for survival of patients with prostate cancer. It may be useful in determining the clinical management of patients with prostate cancer in addition to established prognostic markers.

## Background

Survival in patients with prostate cancer has improved in recent years but prognosis remains poorly understood. It is often difficult to differentiate high risk patients who require potentially curative treatment from low risk patients for whom watchful waiting is sufficient. There is also increasing evidence that radical prostatectomy, with its high iatrogenic morbidity, confers no appreciable survival benefit to watchful waiting in localized disease [1]. Considerable effort has gone into identifying novel genetic and immunological biomarkers for prostate cancer outcomes. However, these remain time consuming and not validated within routine clinical practice [2,3]. Currently, imprecise clinical prognostication is based on readily available tumour related factors, including Prostate Specific Antigen (PSA) levels, Gleason score, surgical margins and pathological stage [4].

There is increasing recognition that systemic inflammation is associated with progression and reduced survival of prostate cancer patients [5,6]. In particular, the systemic inflammatory response, as evidenced by an elevated C-reactive protein (CRP), has been shown to be independently associated with poor prognosis in localised and metastatic prostate cancer [7]. For example, in a retrospective study of 160 patients from the ASCENT (Androgen- Independent Prostate Cancer Study of Calcitriol Enhancing Taxotere) trial, CRP levels appeared to be a predictor of poorer survival [8]. This finding was also shown in another independent dataset of 119 patients with castration-resistant prostate cancer (of whom 57 received docetaxel) enrolled in six phase II clinical trials [9]. These initial findings are limited, however, by the relatively small number of cases and prognostic factors that were considered and adjusted for in the multivariate analysis. Earlier studies on systemic inflammation and prostate cancer survival had smaller sample sizes and follow-up was also limited from 12 to 24 months following diagnosis. In a recent review it has been concluded that CRP might serve as a useful biomarker for urological cancers and that it satisfies the 2001 NIH criteria to be used as a biomarker [10].

More recently, systemic inflammation based prognostic scores such as the modified Glasgow Prognostic Score (mGPS, a combination of C-reactive protein and albumin), have been developed [7] and found to have significant prognostic value in one-year and five-year survival from prostate cancer [11]. However, thes findings from the Glasgow Inflammation Outcome Study (GIOS), failed to account for PSA and comorbidities that would be known to clinicians at the time of diagnosis. Furthermore, earlier study could not examine the relationship between mGPS and long term survival. Therefore the aim of this study was to examine in greater detail the associations between the mGPS and survival in a large mature cohort of patients with prostate cancer and to establish whether it had prognostic significance independent of PSA and comorbidities.

## Methods

Data of prostate cancer patients diagnosed between 2000 and 2006, from the Scottish Cancer Registry, (Scottish Morbidity Record number six (SMR06)) were obtained. Prostate cancer was defined as International Classification of Diseases (ICD), revision 10 code C61. We identified prostate cancer patients in the North Glasgow biochemistry database by extracting records of all patients for whom PSA had been requested. We linked Cancer Registry records to routine biochemistry laboratory records using an indexing method that ensured that patient identifiers and clinical information were never transferred in the same dataset. The linkage was carried out by exact matching of patients’ forename, surname and date of birth, followed by a Soundex phonetic matching algorithm if initial exact matching was unsuccessful. Only data for those patients who had a blood sample taken within a period of six months before or six months after the diagnosis of prostate cancer were included. Out of 8,483 prostate cancer patients diagnosed in the West of Scotland region from 1st January 2000 to 31st December 2006, PSA data were available for 1,861 patients in Glasgow. Of these, patients whose data for C-reactive protein and albumin were available were included in this study. If more than one record was available for a patient within a six month period (before or after diagnosis) then only the record close to the date of diagnosis was used.

The Gleason grading system is known to be associated with prostatic cancer prognosis [12] and was used to describe tumour morphology. Gleason score was extracted from the Scottish Cancer Registry, where available. The information on Gleason score was obtained through prostatic biopsy. The number of hospital in-patient bed days in the period of 10 years up to 1 year preceding diagnosis of prostate cancer were also obtained and used as a crude measure of general pre-existing co-morbidity. In-patient bed days have been previously used as measure of co-morbidity in patients with breast and colorectal cancer in Scotland [13]. Date and cause of death was extracted through cancer registration patient based linkage with National Records of Scotland death records.

Socio-economic status of individuals was assigned by matching their postcode of residence at diagnosis to the Scottish Index of Multiple Deprivation (SIMD) 2006 score. SIMD is an area-based measure of socio-economic circumstances that ranks small geographic areas of Scotland (datazones) from 1 (most deprived) to 6505 (least deprived) using 31 indicators that cover current income, employment, health, education, housing and access [14]. The datazones are further grouped into national quintiles that range from least deprived to the most deprived. The modified Glasgow Prognostic score was constructed as described in Table 1[15]. This study was approved by the West of Scotland Research Ethics Service (WoSRES reference number 11/AL0249).

**Table 1:** The modified Glasgow prognostic score

**Prognostic score**	**Score**
**The modified Glasgow prognostic score**	
C-reactive protein ≤ 10 mg/l	0
C-reactive protein > 10 mg/l and albumin ≥ 35 g/l	1
C-reactive protein > 10 mg/l and albumin < 35 g/l	2

### Statistical analysis

Follow up was from date of incidence of cancer to the date of death or censor date (31st December 2011), whichever came first. Relative survival was used as a measure of cancer patients’ survival. Relative survival has a key advantage over the cause specific survival as it does not rely on the accurate classification of cause of death; instead it provides a measure of total prostate cancer associated excess mortality.

Five and ten year relative survival estimates were made by using age and deprivation specific life tables provided by National Records of Scotland (formerly the General Register Office). These were available until 2009 so for the purposes of this study, the 2009 mortality rates were used for both 2010 and 2011. Relative survival estimates were made by age, deprivation, Gleason score and mGPS, PSA and previous in-patient bed days using the complete and hybrid approach (by STREL and STRS commands in STATA) [16]. The STRS command in STATA implements the Ederer II method by default for the estimation of relative survival; however, we repeated the analyses using both the Ederer I and Hakulinen approaches. All three methods provided identical results, so the results presented in this study are based on the Ederer II Method. Using Poisson regression modelling, the relative excess risk was estimated after adjusting for age, deprivation and Gleason score, PSA and previous in-patient bed days [16]. The lowest category was used as referent for the mGPS and all other categorical covariates. All analyses were conducted using STATA version 11 (StataCorp, College Station, TX, USA). Adherence to the proportional hazards assumption was investigated by plotting smoothed Schoenfeld residuals against time; no violations of the assumption were identified. All statistical tests were two tailed and statistical significance was taken as p < 0.05.

## Results

A total of 744 patients who had a diagnosis of prostate cancer, and had biochemistry data within six months before or after diagnosis, were included in this study. The majority of patients, 578 (78%), were aged 65 or over. Thirty five percent of patients (n = 262) had high Gleason score (Gleason 8–10), 21.9% had Gleason score missing (n = 163) and nearly half of the cohort (n = 362, 49%) had PSA greater than 20ug/l. More than a third of patients (n = 272, 37%) lived in the most socio-economically deprived areas while only 18% lived in the most affluent areas. The median follow-up from the cancer diagnosis was 4.11 years, and maximum 11.9 years.

Patients with an elevated mGPS (mGPS 1 and 2) were significantly more likely to be 75 years or older (p = 0.014) and have either high Gleason score disease (Gleason 8–10) or unknown Gleason (p < 0.001) but there was no association with socioeconomic circumstances based on SIMD (p = 0.219) – Table 2. Patients with an elevated mGPS were significantly more likely to have raised PSA (PSA > 20 ug/l) and less likely to have higher previous inpatients bed days (p-value 0.022).

**Table 2:** Baseline characteristics of patients with prostate cancer based on mGPS categories

	**The modified Glasgow prognostic score (mGPS)**			**P-value**
	**mGPS = 0**	**mGPS = 1**	**mGPS = 2**	
	**Patients, n (%)**	**Patients, n (%)**	**Patients, n (%)**	
Age at incidence (years)				
Age < 65	88 (25.29)	71 (21.78)	7 (10.00)	0.014
Age 65-74	127 (36.49)	112 (34.36)	22 (31.43)	
Age ≥ 75	133 (38.22)	143 (43.87)	41 (58.57)	
Gleason score				
Gleason < 7	95 (27.30)	67 (20.55)	15 (21.43)	<0.001
Gleason = 7	85 (24.43)	51 (15.64)	6 (8.57)	
Gleason 8-10	124 (35.63)	113 (34.66)	25 (35.71)	
Unknown Gleason	44 (12.64)	95 (29.14)	24 (34.29)	
SIMD 2006, Quintiles				
1 (most deprived)	117 (33.62)	128 (39.26)	27 (38.57)	0.219
2	71 (20.40)	70 (21.47)	9 (12.86)	
3	46 (13.22)	45 (13.80)	9 (12.86)	
4	43 (12.36)	28 (8.59)	13 (18.57)	
5 (least deprived)	71 (20.40)	55 (16.87)	12 (17.14)	
Prostate specific antigen (ug/l)				
PSA < 10	140 (40.35)	99 (30.46)	26 (37.14)	<0.001
PSA 10-20	69 (19.88)	42 (12.92)	4 (5.71)	
PSA > 20	138 (39.77)	184 (56.62)	40 (57.14)	
Previous inpatient bed days				
0	200 (57.47)	174 (53.37)	30 (42.86)	0.022
1-7	73 (20.98)	65 (19.94)	17 (24.29)	
8-28	55 (15.80)	64 (19.63)	11 (15.71)	
29+	20 (5.75)	23 (7.06)	12 (17.14)	

Increasing age, Gleason score, PSA and previous inpatient bed days were associated with poorer 5 and 10 year relative survival (Table 3). Decreasing deprivation was associated with better 5 and 10 year relative survival. On multivariate analysis, increasing age, Gleason score, PSA > 20 ug/l, previous inpatients bed days >28 and mGPS were the major predictors of relative excess risk of death at 5 and 10 years (Table 3). Compared with patients with an mGPS of 0, patients with an mGPS of 1 and 2 had higher risks of death in the five years following diagnosis (RER 1.84, 95% CI 1.33-2.55, p <0.001 and RER 3.57, 2.31-5.25, p < 0.001, respectively) which was independent of age, Gleason score, SIMD, PSA and previous inpatient bed days. Similarly, 10 year mortality was raised in patients with mGPS of 1 and 2 (RER 1.87. 95% 1.37-2.55, p <0.001 and RER 3.42, 95% 2.25-5.21, p < 0.001, respectively) after adjusting for other factors (Table 3).

**Table 3:** The relationship between patient characteristics and five and ten year relative survival and relative excess risk of death of patients with prostate cancer

	**Five year survival and excess risk of death**		**P-value**	**Ten year survival and excess risk of death**		**P-value**
	**5-year relative survival**	Relative excess risk (95% CI) *		**10-year relative survival**	Relative excess risk (95% CI)*	
Modified Glasgow prognostic score						
0	74.8 (67.9-81.3)	1		51.4 (42.8-60.1)	1	
1	48.3 (41.1-55.1)	1.84 (1.33-2.55)	<0.001	22.4 (16.6-29.1)	1.87 (1.37-2.55)	<0.001
2	32.6 (19.8-47.3)	3.57 (2.31-5.52)	<0.001	18.8 (7.6-36.1)	3.42 (2.25-5.21)	<0.001
Age at incidence (years)						
Age < 65	73.3 (65.1-80.2)	1		47.4 (37.6-56.9)	1	
Age 65-74	58.4 (50.9-65.6)	1.69 (1.15-2.49)	0.008	36.1 (28.3-44.3)	1.49 (1.05-2.12)	0.026
Age ≥ 75	51.6 (43.2-60.3)	1.92 (1.31-2.80)	0.001	24.4 (16.8-33.7)	1.69 (1.20-2.40)	0.003
Gleason score						
Gleason < 7	96.6 (87.6-100)	1		68.9 (56.1-81.1)	1	
Gleason = 7	77.0 (66.4-86.2)	2.96 (1.19-7.37)	0.020	44.3 (32.5-56.5)	2.74 (1.28-5.86)	0.010
Gleason 8-10	50.0 (42.2-58.0)	7.18 (3.12-16.50)	<0.001	25.1 (17.9-33.3)	5.55 (2.75-11.20)	<0.001
Unknown Gleason	16.3 (10.4-23.7)	16.27 (7.09-37.34)	<0.001	7.5 (3.2-14.7)	12.44 (6.12-25.29)	<0.001
SIMD 2006, Quintiles						
1 (most deprived)	49.9 (42.8-56.9)	1		27.8 (20.9-35.5)	1	
2	51.0 (40.9-61.1)	1.24 (0.88-1.73)	0.221	25.4 (16.5-36.0)	1.28 (0.92-1.78)	0.146
3	70.4 (56.6-82.9)	0.80 (0.49-1.30)	0.364	35.2 (22.4-49.9)	0.91 (0.58-1.42)	0.682
4	74.4 (59.2-87.9)	0.81 (0.48-1.36)	0.419	49.0 (32.1-67.3)	0.93 (0.58-1.49)	0.761
5 (least deprived)	71.9 (59.4-83.6)	0.80 (0.51-1.24)	0.312	58.7 (42.2-76.2)	0.81 (0.53-1.23)	0.319
Prostate specific antigen (ug/l)						
PSA < 10	78.0 (70.4-84.8)	1		58.2 (48.4-67.9)	1	
PSA 10-20	73.1 (60.6-84.2)	0.78 (0.44-1.38)	0.396	45.4 (31.4-60.3)	0.78 (0.44-1.39)	0.409
PSA > 20	40.4 (34.0-47.0)	1.47 (1.01-2.14)	0.041	15.4 (10.7-21.2)	1.82 (1.26-2.63)	0.002
Previous inpatient bed days						
0	60.8 (54.5-66.9)	1		36.7 (30.3-44.0)		
1-7	70.0 (59.2-79.9)	0.78 (0.53-1.15)	0.205	41.5 (29.7-54.2)	0.75 (0.51-1.09)	0.141
8-28	52.7 (41.3-64.0)	1.07 (0.74-1.55)	0.708	26.3 (16.2-38.7)	1.12 (0.79-1.59)	0.530
29+	33.7 (19.7-49.5)	1.65 (1.09-2.51)	0.018	19.2 (7.1-38.6)	1.64 (1.08-2.47)	0.019

*Multivariate model included all the co-variates presented in the table.

When the analysis was stratified based on Gleason score and PSA level, we observed a significant association between mGPS and risk of death within ten years of diagnosis with PSA < 10 ug/l group (RER 9.65, 95% CI 3.13-29.75, p for trend <0.001), PSA 10-20ug/l category (RER 2.50, 95% CI 0.20-31.07, p for trend 0.088) and those with PSA > 20 ug/l (RER 5.01, 95% CI 3.05-8.22, p for trend 0.001) after adjustment for age, socioeconomic circumstances and previous inpatient bed days (Table 4). In grade-specific analysis, we observed a significant association between the mGPS and risk of death within 10 years at all grades of disease: low grade (RER 20.46, 95% CI 3.43-121.97, p for trend <0.001), intermediate grade (RER 2.25, 95% CI 0.31-16.08, p for trend 0.003), high grade (RER 1.88, 95% CI0.98-3.61, p for trend 0.035) and unknown grade (RER 1.97, 95% CI 1.03-3.73) after adjustment for other factors (Table 4).

**Table 4:** Relative excess risk of death of prostate cancer patients based on Gleason score and PSA categories

	**Modified Glasgow prognostic score**			***P-value for trend***
	**0**	**1**	**2**	
**Gleason score-specific analysis**				
***Gleason < 7***				
Relative excess risk (95% CI)^a^	reference	1.66 (0.15-18.65)	46.04 (4.59-461.70)	0.013
Relative excess risk (95% CI)^b^	reference	3.26 (0.67-15.89)	20.46 (3.43-121.97)	0.002
***Gleason = 7***				
Relative excess risk (95% CI)^a^	reference	3.06 (1.28-7.35)	2.19 (0.25-19.20)	0.021
Relative excess risk (95% CI)^b^	reference	3.99 (1.78-8.97)	2.25 (0.31-16.08)	0.003
***Gleason 8-10***				
Relative excess risk (95% CI)^a^	reference	2.09 (1.29-3.37)	5.27 (2.73-10.22)	<0.001
Relative excess risk (95% CI)^b^	reference	2.04 (1.30-3.22)	5.64 (3.00-10.59)	<0.001
***Unknown Gleason***				
Relative excess risk (95% CI)^a^	reference	1.55 (0.98-2.46)	2.18 (1.13-4.22)	0.015
Relative excess risk (95% CI)^b^	reference	1.51 (0.96-2.37)	1.88 (0.98-3.61)	0.035
**PSA-specific analysis**				
***PSA < 10ug/l***				
Relative excess risk (95% CI)^a^	reference	3.43 (1.37-8.59)	8.29 (2.76-24.87)	<0.001
Relative excess risk (95% CI)^b^	reference	3.86 (1.48-10.03)	9.65 (3.13-29.75)	<0.001
***PSA 10-20ug/l***				
Relative excess risk (95% CI)^a^	reference	1.87 (0.63-5.58)	3.65 (0.47-28.49)	0.163
Relative excess risk (95% CI)^b^	reference	2.54 (0.90-7.18)	2.50 (0.20-31.07)	0.088
***PSA > 20ug/l***				
Relative excess risk (95% CI)^a^	reference	2.20 (1.50-3.24)	5.08 (3.03-8.55)	<0.001
Relative excess risk (95% CI)^b^	reference	2.25 (1.57-3.20)	5.01 (3.05-8.22)	<0.001

All estimates were presented after adjusted for age, deprivation and inpatient bed days. a = five-year relative survival, b = 10-year relative survival.

After excluding deaths in first 12 months following diagnosis (n = 149) to minimise the effects of “reverse causality”, elevated mGPS showed an increased risk of death at five (RER 2.43, 95% CI 1.23-4.79, p-value 0.011) and ten years (RER 2.42, 95% CI 1.29-4.58, p-value 0.006) after adjusting for age, Gleason score, socioeconomic circumstances, PSA and inpatient bed days (Table 5).

**Table 5:** Five and ten year conditional relative survival and relative excess risk of death of prostate cancer patients

	**Five year survival and excess risk of death**		**P-value**	**Ten year survival and excess risk of death**		**P-value**
	**5-year relative survival**			**Relative excess risk (95%CI)**	**10-year relative survival**	**Relative excess risk (95%CI)**	
Modified Glasgow prognostic score						
0	83.0 (75.8-89.5)	1		81.7 (65.6-94.2)	1	
1	64.1 (55.7-72.0)	1.66 (1.12-2.46)	0.012	43.2 (32.0-55.5)	1.75 (1.21-2.53)	0.003
2	56.9 (35.6-77.3)	2.43 (1.23-4.79)	0.011	38.8 (16.6-67.0)	2.42 (1.29-4.58)	0.006

Estimates adjusted for age, Gleason score, socioeconomic circumstances, PSA and inpatient bed days. Survival and risk estimates taken after excluding the deaths in first 12 months following the diagnosis.

**Figure 1:**
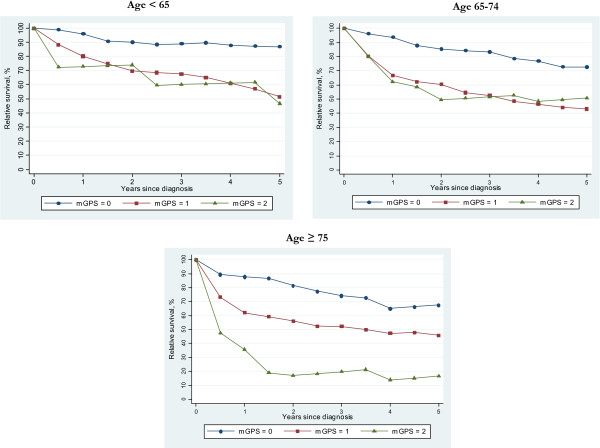
The modified Glasgow prognostic score and survival based on age categorie.

Figure 1 shows the age-specific relative survival of prostate cancer patients based on the mGPS categories. Raised level of mGPS (1 and 2), showed significantly poorer survival in all age groups with particularly worse survival in the oldest group (age ≥ 75). There was no convincing difference in mortality between patients with mGPS scores of 1 and 2 in patients under 75 years of age.

## Discussion

The results of the present study indicate that a raised level of mGPS is associated with poorer short and long term survival in men with prostate cancer. This relationship was independent of age at diagnosis, socio-economic circumstances, Gleason score, PSA level and previous in-patient bed days. These findings are consistent with earlier observations from the Glasgow Inflammation Outcome Study, where the mGPS was compared with Neutrophil Lymphocyte Ratio and demonstrated significant prognostic value [11]. The prognostic value of mGPS remained consistent even after excluding deaths in the first 12 months after diagnosis, which suggest that disease stage is unlikely to explain the survival differences between mGPS categories.

We observed 40% and 22% lower 5-year and 10-year relative survival respectively, among those with raised modified Glasgow Prognostic Score (mGPS = 2) compared to the normal (mGPS = 0) following diagnosis of prostate cancer. In the present study, patients with raised mGPS were significantly more likely to have unknown Gleason score and less likely to have low grade disease compared with the mGPS of 0. Similarly, patients with raised mGPS (mGPS = 2) were significantly more likely to have PSA > 20ug/l. In Gleason score specific analysis, a raised mGPS had significant associations with excess risk of death among patients regardless of disease grading. The largest effect of mGPS was seen in patients with low grade prostate cancer (Gleason < 7), i.e. men with raised mGPS (mGPS = 2) were 46 and 20 times more likely to die in the first five and ten years following diagnosis compared to patients with a mGPS of 0. The large effect and wide confidence interval in this category may be due to the small number of cases with low Gleason score and raised mGPS (n = 15), of whom 11 died during ten years follow up.

In PSA specific analysis, patients with raised mGPS were significantly more likely to die in five and ten years in both, PSA < 10ug/l and PSA > 20ug/l categories. Although there was no significant association between mGPS and survival in the intermediate PSA category (PSA 10-20ug/l), this could have been due to the small number of cases in intermediate PSA category with raised mGPS (n = 4).

In the present study, patients with raised mGPS had poorer five and ten year survival even when the deaths in the first 12 months were excluded from the analysis. This was based on the assumption that patients with metastatic disease may have raised level of inflammatory markers and the overall effect of mGPS may be driven by the advance stage disease among men with the raised mGPS. Exclusion of early deaths from analysis did not change the prognostic value of the mGPS, this suggest that differential distribution of metastatic disease between mGPS categories is unlikely to explain the prognostic significance of mGPS. Furthermore, previous studies have shown systemic inflammation to be associated with survival, independent of disease stage, for gastroesophageal, colorectal (including those with liver metastases), renal, breast and prostate cancers [17-19] however, the findings of earlier prostate cancer study are based on smaller sample (n = 62) [17].

Additionally, the raised mGPS (1 and 2) has shown poorer survival in all age groups. This is of particular interest in the younger age group (<65 years) where most uncertainty lies about the management of disease and treatment decisions are made on the basis of individual’s age, fitness, comorbidity, PSA, Gleason score and disease stage. Novel genetic and immunological biomarkers have been identified but these, to date, have not been incorporated into routine clinical practice [2,3]. The results of the present study further strengthen the earlier observations that systemic inflammation is of clinical importance and suggest the routine use of the mGPS may be a cost effective, readily available tool for risk stratification in patients with prostate cancer.

Strengths of our study include its large sample size, inclusion of information on PSA and Gleason score and a fairly long follow-up to determine the effect of systemic inflammation on short and long term survival. However, our study has limitations. First, patients were selected on the basis of availability of PSA, C-reactive protein and albumin, therefore this cohort of patients might not be representative of all the prostate cancer patients diagnosed and treated in the area. Second, the reason why these patients were tested for C-reactive protein remains unclear and there is a possibility that they might have had concurrent morbidity for which they were clinically investigated. However, this is unlikely to have had a major effect on our results, as we adjusted for background mortality as well as the previous inpatient bed days from ten years to one year prior to the diagnosis of prostate cancer. The value of mGPS between different treatment groups need to be evalued in future work and further work is also required to investigate this relationship in a larger, representative sample of prostate cancer patients including information on disease stage.

## Conclusion

The mGPS is an objective prognostic marker for survival in prostate cancer patients and has additional value to other conventional, routinely available information. Prospective studies are required to validate our results and to test the clinical utility of mGPS in the clinical management of prostate cancer.

## Competing interests

All authors declare that they have no competing of interest.

## Authors’ contributions

KS (Kashif Shafique) and DSM designed the study. KS (Kashif Shafique) carried out statistical analyses. KS (Karen Smith) and BS carried out data extraction and linkage. All authors contributed to interpreting the results; KS (Kashif Shafique) wrote the initial draft. KS, DCM and DSM revised and finalised the manuscript; all authors saw and approved the final manuscript.

## Pre-publication history

The pre-publication history for this paper can be accessed here:

http://www.biomedcentral.com/1471-2407/13/292/prepub
